# Sex Differences in Long-Term Cardiovascular Outcomes and Mortality After COVID-19 Hospitalization During Alpha, Delta and Omicron Waves

**DOI:** 10.3390/jcm13226636

**Published:** 2024-11-05

**Authors:** Kristen Kopp, Lukas J. Motloch, Michael Lichtenauer, Elke Boxhammer, Uta C. Hoppe, Alexander E. Berezin, Diana Gareeva, Irina Lakman, Alexander Agapitov, Liana Sadikova, Venera Timiryanova, Paruir Davtyan, Elena Badykova, Naufal Zagidullin

**Affiliations:** 1 University Clinic for Internal Medicine II, Paracelsus Medical University, Muellner Hauptstrasse 48, 5020 Salzburg, Austriam.lichtenauer@salk.at (M.L.); e.boxhammer@salk.at (E.B.); u.hoppe@salk.at (U.C.H.); aeberezin@gmail.com (A.E.B.); 2 Department of Internal Medicine II, Salzkammergut Klinikum, OÖG, 4840 Vöcklabruck, Austria; 3 Department of Cardiology, Kepler University Hospital, Medical Faculty, Johannes Kepler University, 4040 Linz, Austria; 4 Department of Internal Diseases, Bashkir State Medical University, Lenin Str., 3, 450008 Ufa, Russia; danika09@mail.ru (D.G.); i@pdavtjan.ru (P.D.); lnurova@mail.ru (E.B.); zsnaufal@gmail.com (N.Z.); 5 Scientific Laboratory for the Socio-Economic Region Problems Investigation, Ufa University of Science and Technology, Zaki Validi Str. 32, 450076 Ufa, Russia; lackmania@mail.ru (I.L.);

**Keywords:** COVID-19, sex, cardiovascular outcomes, cardiovascular mortality, follow-up

## Abstract

**Background:** Increased mortality and occurrence of cardiovascular (CV) outcomes during hospitalization and in short-term follow-up for moderate to severe SARS-CoV-2 infection have been associated with male sex, yet data regarding long-term outcomes by sex and COVID-19 variant (Alpha, Delta, and Omicron) are limited. **Methods**: This prospective study of 4882 patients examines potential differences by sex in the occurrence of primary combined cardiovascular outcomes (CV death, CV hospitalization, myocardial infarction (MI), stroke, pulmonary embolism) as well as secondary outcomes (CV death, cardiovascular hospitalizations, myocardial infarction, stroke, pulmonary embolism) at 18-month follow-up after urgent hospitalization for SARS-CoV-2-associated pneumonia, as well as evaluating for differences during the three COVID-19 waves. Survival rate was analyzed for the entire cohort by sex and SARS-CoV-2 variant and adjusted for age using the multiple Kaplan–Meier method. To compare survival in groups of men and women for each wave, the Gehan–Wilcoxon test was applied with significance *p* < 0.05. Univariate Cox proportional hazards models were used to search for potential risk factors of CV death at 18-months follow-up separately for men and women in each COVID-19 wave. **Results**: Men had significantly higher 18-month CV mortality compared to women in the Delta wave (6.13% men vs. 3.62% women, *p* = 0.017). Although men had higher percentages of all other CV endpoints (excepting pulmonary embolism) at follow-up during the Delta wave, none were significant compared with women, except for the combined CV endpoint (16.87% men vs. 12.61% women, *p* = 0.017). No significant differences by sex in CV outcomes were seen during the Alpha and Omicron variants. Discrepancies in CV outcomes in demographical data and concomitant disease between the COVID-19 variants of concern existed. **Conclusions:** Higher male mortality and higher but non-significant incidences of CV outcomes occurred during the Delta wave of the COVID-19 pandemic, with the lowest incidence of CV outcomes observed during the Omicron variant.

## 1. Introduction

Critical SARS-CoV-2 infection predominantly affects the lower respiratory tract and has been associated with up to 30% increased mortality risk among patients admitted to critical care units, a risk persisting among the critically ill during the Alpha and Delta variants, yet present but diminishing with emergence of the Omicron variant [1]. In addition to respiratory manifestations, such as interstitial pneumonia and acute respiratory stress syndrome, other associated complications in the acute phase have been observed. They include coagulopathies, venous and arterial thrombosis [2], transient kidney [3] and liver injuries [4], neurological conditions [5], as well as an array of cardiovascular (CV) complications such as acute coronary syndromes, acute myocardial injury without obstructive coronary artery disease (CAD), myocarditis, arrhythmias, pericardial effusion, and acute/acutely decompensated heart failure, including cardiogenic shock [6].

While the majority of patients will spontaneously recover, or recover following acute-phase management of the infection, more than half of COVID-19 survivors will experience post-acute COVID-19 syndrome (PACS), suffering from a diverse set of symptoms requiring inter-/multiple-disciplinary short- or long-term management [7,8]. Common post-infection sequelae among patients with mild-to-severe infection can range from prolong fatigue, dyspnea, persistence dry cough, palpitations, chest pain/discomfort, headache, and decreased cognitive status to olfactory dysfunction [9,10]. However, respiratory and lung function sequelae among patients hospitalized for SARS-CoV-2 infection may persist and be more severe. COVID-19 can damage alveolar epithelial and endothelial cells with secondary fibroblast proliferation, potentially causing interstitial lung fibrosis or pulmonary hypertension through vascular and alveolar remodeling and thrombotic complications [11].

A spectrum of cardiovascular sequelae and events in COVID-19 patients post-discharge have been described in the early acute phase following infection (defined as 21 or 30 days from the start of infection by study) and in the post-acute phase (up to 1-year post-infection) [10,12]. A large UK Biobank study showed that patients in the acute phase following COVID-19 infection had roughly four times higher risk for developing major cardiovascular disease (CVD), defined as composite heart failure, stroke, and/or coronary heart disease events, and were at nearly 81 times higher risk of all-cause mortality compared to uninfected historical and contemporary control groups [12]. In the post-acute phase (more than 21 days after infection), authors observed that risk persisted, noting a 50% increased risk of major CVD and 5 times the risk of all-cause mortality compared to non-infected historical and contemporary control groups [12]. To note, more severe COVID-19 infection correlated with higher risks of CVD and mortality post-infection compared to a less severe disease course, although patients with less severe disease still exhibited increased risk of CV outcomes and mortality when compared to uninfected groups [12]. A large multinational study of nearly 50,000 hospitalized COVID-19 patients confirmed these findings, observing that major adverse CV events (MACEs) occurred in 17.8% of patients with severe infection, and that patients with in-hospital MACE had higher 28-day and 90-day mortality compared to those that did not (63.1% vs. 35.6%, *p* < 0.001; 69.9% vs. 37.8%, *p* < 0.001, respectively) [13].

A large longitudinal population health study of 153,760 US veterans (predominantly Caucasian, 89% males/11% females) following acute COVID-19 infection observed a persistent and substantial increased risk and burden of MACEs, all-cause mortality, and any CV outcome among infected individuals compared to over 5 million non-infected contemporary and historical controls during 12 months follow-up [14].

The risk of composite ischemic disease outcomes and corresponding burden was increased (HR = 1.66, 1.52, 1.80) with a burden of 7.28 per 1000 persons (5.80, 8.88) [14]. Risk of developing heart failure at 1-year post-infection was pronounced (HR = 1.72 (1.65, 1.80) with excess burden of 11.61 (10.47, 12.78). Also described was an increased risk of cerebrovascular disorders and burden, including increased risk of stroke (Hazard ratio (HR) = 1.52 (1.43, 1.62)) and burden 4.03 (33.32, 4.79) per 1000 persons at 12 months (95% CI), as well as risk of transient ischemic attacks (TIA) (HR = 1.49) and burden 1.84 (1.38, 2.34) among patients surviving the first 30 days post-infection [14].

The authors concluded that the risk of incident CV disease extends well beyond the acute phase of infection [14]. Of note, the results of the study showed male bias due to the predominantly male demographic.

In a subsequent study of 77,364 women and minority veterans from the US Veterans Health Administration database, women infected with COVID-19 (*n* = 8308) also exhibited a 4 times higher mortality risk at 60 days post-infection than non-infected female controls, a risk that persisted across all ethnic groups. However, while infected females showed significantly increased risk of developing myocardial ischemia during the first 60 days post-infection, the risk of developing composite CV events (ischemic and hemorrhagic stroke, early MI, and heart failure) and developing new heart disease conditions at 60-days post-infection was actually lower compared to non-infected female counterparts [15]. Authors argued that the discrepancy may have been caused by the younger age of females who tested positive, stating that longer post-infection follow-up of women is warranted [15].

CVD pathogenesis in both the acute phase of COVID-19 as well as post-acute COVID-19 may impact short- and long-term survival, with plausible dependence on gender.

The literature describes several risk factors associated with susceptibility for infection and severity and mortality in COVID-19 disease progression. These include older age (defined as age > 65, 70, or 75 years, according to this study), male sex, and presence of comorbidities such as severe obesity (BMI ≥ 40), hypertension, diabetes mellitus, malignancy, COPD, chronic kidney disease, and cardiovascular diseases [16,17,18,19,20]. According to some publications, smoking or blood type A are additional factors that may increase the risk for severe outcomes [17,21,22]. While comorbidities place patients at a higher risk of dying from a COVID-19 infection, Global Health 50/50 data highlight the association of age and (male) sex on differences in COVID-19 infection and mortality rates [23]. During hospitalization for COVID-19, males have a greater risk of death after acute infection, and females show 40% greater ICU survival than males independent of age [24,25]. Males not only have a significantly elevated hazard of death, but males experience a higher severity of illness, more often experiencing cardiac arrest and pulmonary embolism, and more often receive treatment with ECMO [24].

While males have greater severity of disease and higher mortality rates during acute infection, findings regarding sex differences in the occurrence of post-COVID-19 sequelae are more ambiguous. In contrast to the US Veterans study, an Italian study showed female sex was an independent predictor of MACCE (CV death, myocardial infarction, stroke, pulmonary embolism, acute heart failure, or hospitalization for all cardiac causes) with a 2.6-fold increased risk of MACCE observed in females compared to males at six months follow-up post-infection [26]. A literature review also described sex-aggregated differences in post-COVID-19 sequelae, where musculoskeletal and respiratory sequelae were more common in females and acute renal injury was more common in males within 4 weeks post-infection. Point estimates for cardiovascular sequelae favored females (OR = 0.95; 95% CI 0.88, 1.03) in that study [27]. Concerning long-Covid syndrome, incidences were higher in females, with the odds of ENT, GI, psychiatric/mood, neurological, and dermatological disorders significantly higher for females compared to males, while males showed higher odds of experiencing endocrine or renal disorders [27]. It is important to note that the size of female cohorts and reporting of sex-aggregated data is not always sufficient or standardized, and sex-aggregated data in post-COVID-19 follow-up are limited [28]. Indeed, in the above review of 4346 publications, only 23 studies contained sex-disaggregated data regarding COVID-19 sequelae, despite a United Nations global mandate issued in 2020 urging for characterization of the differential impact of sex on COVID-19 detection and clinical management [27].

Most data sources stem from the earlier stages of the pandemic, with data gathered in 2020. Several studies have examined in-hospital and all-cause mortality in the later Alpha, Delta, and Omicron waves, but the literature is sparse with respect to short- and long-term outcomes following each of these waves, and, to our knowledge, none have examined potential differences in long-term outcomes by sex for all three waves. With respect to in-hospital mortality by COVID waves, the multinational EuCARE project evaluated 28-day in-hospital mortality rates using cumulative incidence function and sub-distribution hazard ratios (SHR) adjusted for age, sex, calendar time, and comorbidities in 38,585 patients [29]. Among patients aged ≥ 70 years, a decrease in in-hospital mortality could be observed over time. The adjusted SHR (95% CI) was 1.66 (1.17–2.36) for Alpha versus Omicron, 1.66 (1.29–2.13) for Delta versus Omicron, and for unvaccinated persons, 1.21 (0.68–2.17) and 1.21 (0.81–1.82), respectively. Among Omicron sub-lineages, the SHR for earlier BA.1 was 1.92 (1.43–2.58) compared to BA.2 and 1.52 compared to BA.5 [29]. Authors noted that the decrease in in-hospital mortality was potentially affected not only by vaccination or developed immunity through previous infections but potentially may be attributed to the differences in virulence between the SARS-CoV-2 variants [29].

Given the paucity of previous studies with sex-aggregated data and limited data regarding long-term follow-up post-COVID-19 infection by sex, our study aims to clearly elucidate (1) any sex-specific differences in the occurrence of adverse CVD outcomes and mortality at 18 months post-COVID-19 hospitalization and (2) further evaluate for any potential sex differences in CV outcomes and mortality occurring among patients infected during each of the Alpha, Delta, and Omicron SARS-CoV-2 waves.

## 2. Materials and Methods

### 2.1. Study Cohort, Data Collection and Analyses

In total, 4882 patients hospitalized with moderate to severe SARS-CoV-2-associated pneumonia between 16 April 2020, and 17 March 2022, during the first, second, and third COVID-19 waves (Alpha, *n* = 2275, Delta *n* = 1721, Omicron *n* = 886) were included in this prospective, non-randomized, single-center study. All included patients were hospitalized for treatment at the COVID-Hospital of Bashkir State Medical University (Bashkir State, Russian Federation) with SARS-CoV-2 infection confirmed by a positive COVID-19 polymerase chain reaction (PCR) test result and/or clinically/radiologically confirmed SARS-CoV-2 infection (i.e., as ground-glass opacity and/or crazy paving on chest computed tomography (CT) scan) and with follow-up data available at 18 months. In total, 373 patients were excluded, including 9 patients with missing data (Alpha group) and 364 patients who died before hospital discharge (146 Alpha group, 107 Delta group, and 111 Omicron group).

All eligible patients were followed for 18 months to capture mortality and the occurrence of long-term cardiovascular (CV) outcomes. Survival status and capture of CV outcomes at 18 months was recorded using a remote data capture system “ProMed” (Program for Medical Cases Monitoring) and patients were contacted by phone or assessed in ambulatory or in-hospital visits in case of events or readmission.

Demographic variables captured in the analysis included age, sex, Body Mass Index (BMI), concomitant CVD, coronary heart disease (CHD), arterial hypertension (AH), diabetes mellitus (DM), chronic kidney disease (CKD), prior myocardial infarction, acute/chronic heart failure, prior stroke, chronic obstructive pulmonary disease (COPD). CV outcomes were defined as CV death, CV hospitalizations, myocardial infarction (MI), stroke, pulmonary embolism, and a combined CV endpoint (CV death, CV hospitalization, MI, stroke, pulmonary embolism). The primary endpoint was CV mortality at 18 months post-discharge for hospitalization for COVID-19-associated pneumonia.

COVID-19 waves were defined as Alpha, with patients infected 16 April 2020–31 December 2020, Delta, with patients infected 1 January 2021–31 December 2021, and Omicron, with patients infected 1 January 2022–9 March 2022. The COVID-19 variant was defined according to random but regularly assessed virus identification in nasal smear in the local territorial biological laboratory. Vaccination status was not obtained and this is recognized as a study limitation.

### 2.2. Ethics Declaration

The study received prior approval by the local ethics commission of the Bashkir State Medical University (N9 from 17 December 2021) and was performed according to the principles of Good Clinical Practice and the Declaration of Helsinki. All patients signed informed consent at the time of hospitalization, permitting the capture and use of data, as well as further contact.

### 2.3. Statistical Analysis

The cohort was divided into two groups by sex. The Mann–Whitney test for paired comparisons between men and women and the Kruskal–Wallis test for comparisons of indicators in three groups in three waves of COVID-19 were used to conduct intergroup comparisons of continuous characteristics (body mass index (BMI) and age). Following the Shapiro–Wilk test, nonparametric tests were chosen because continuous traits were not normally distributed (*p* < 0.05). For categorical characteristics, comparisons were made using the Xi-square test for paired comparisons and for comparisons of three groups, and Yates’ correction was applied where necessary. In all criteria, the differences were considered significant at *p*-level < 0.05.

The multiple Kaplan–Meier method was used to analyze survival curves, and for endpoint cardiovascular mortality, those deceased during the follow-up period from other causes were censored. To compare survival in groups of men and women for each wave of COVID-19, the Gehan–Wilcoxon test was used, in which the significance level was taken at *p* < 0.05.

To identify predictors of mortality risk from CVD in the long-term period (1.5 years) after hospitalization for COVID-19-associated pneumonia, multivariate Cox proportional hazards models were constructed separately for the Alpha, Delta, and Omicron waves. The significance of the overall model was estimated by likelihood ratio (LR) test and the coefficient of concordance. The modeling results were interpreted on the calculation of hazard of rate (HR) of CV death in the presence of a corresponding risk predictor, a confidence interval with a reliability of 95% using the Greenwood formula (CI 95%), and the corresponding *p*-level of significance.

R and the “survival”, “stats”, and “survMisc” libraries (Chris Dardis (2013). survMisc: Miscellaneous Functions for Survival Data. R package version 0.5.6, https://cran.r-project.org/web/packages/survMisc accessed on 11 July 2024) were used for statistical calculation.

## 3. Results

### Demographics and Characteristics

The main clinical-demographic characteristics of the total patient population according to COVID-19 variants are found in Table 1. Clinical-demographic characteristics are presented by sex in Table 2 and Table 3.

Table 2 shows further demographics by sex, BMI, and age during each COVID-19 wave. A significantly higher number of women in the total group (3032 women versus 1850 men) and in each of the three COVID waves (Alpha 1298 women (57.1%)/977 men (42.9%); Delta 1134 women (65.9%)/587 men (34.1%); Omicron 600 women (76.7%)/286 men (32.3%)) were hospitalized with SARS-CoV-2-associated pneumonia. Median age in the total group was 59.2 years and the only significant difference in age was observed during the Alpha wave (women median 58.22 years (Q1 50, Q3 67) compared to men median 56 years (44, 66)). Patients in the Alpha cohort were significantly younger than in the Omicron cohort. The median BMI was 27.5, overweight, with no significant differences between men and women in the total cohort or during each COVID-wave (27.76, 27.68 men, 28.44, 28.04 women, Alpha and Delta, respectively), although a non-significant decrease in BMI was seen for both sexes during the Omicron wave (24.17 men, 25.43 women).

Table 3 shows the presence of concomitant diseases by sex and COVID-19 waves. Men had a significantly higher presence of prior coronary heart disease (CHD), prior MI, and COPD during the Alpha wave. In comparison, women had a higher presence of arterial hypertension and diabetes mellitus during the Alpha wave. During the Delta wave, men showed a significantly higher presence of prior CHD, prior MI, and prior stroke compared to women. During Omicron, the presence of heart failure and prior stroke was significantly higher in men compared to women.

Arterial hypertension (AH) was the most common CV risk factor affecting the highest percentages of patients during all variants, ranging in males from 32.96% (Alpha) to 54.41% (Delta) and in women from 39% (Omicron) to 57.23% (Delta), showing highest incidences during the Delta wave. Coronary heart disease was the next most common comorbidity and ranged in males from 11.99% (Omicron) to 22.32% (Delta) and in women from 7.09% (Alpha) to 17.37% (Delta). Again, its incidence was highest during the Delta wave for both sexes.

After performing Fischer’s exact test within the category sex, the proportions of women with arterial hypertension, chronic kidney disease, coronary heart disease, heart failure, and prior stroke differed significantly between the waves (see Table 3 for 3 waves comparison). The same finding was observed in men: the proportions with the comorbidities hypertension, chronic kidney disease, CHD, heart failure, and prior stroke also differed significantly between the waves.

When examining the cardiovascular endpoints, CV death during 18-month follow-up occurred in 2.48% of all patients, but CV mortality rates differed significantly between waves, with Delta showing a significantly higher incidence of CV mortality in long-term follow-up (4.47%) compared to Alpha (1.76%) and Omicron waves (0.45%) *p* < 0.001 (see Table 4). Concerning other CV endpoints, CV hospitalization was significantly increased during the Delta (10.17%) and Alpha (9.14%) waves compared to the Omicron wave (4.92%), *p* < 0.001. Stroke occurred in just 0.66% of all patients during the 18-month follow-up yet was significantly increased in the Delta wave (1.1%) compared to the Alpha (0.31%) and Omicron (0.68%) waves, *p* = 0.009. The combined CV endpoint was observed in 10.1% of all patients and was significantly increased during the Delta (14.6%) compared to Alpha (7.17%) and Omicron (9.93%) waves, *p* < 0.001.

Table 5 compares CV endpoints in COVID-19 patients at 18 months post-hospitalization by sex and variant. When examining CV mortality by waves according to sex, men had a significantly higher 18-month CV mortality rate in the Delta wave compared to women (6.13% men vs. 3.62% women, *p* = 0.017). Although men had higher percentages of all other CV endpoints (excepting PE) in follow-up during the Delta wave, none were significant compared with women, with the exception of the combined endpoint (16.87% men vs. 12.61% women, *p* = 0.017), which was likely driven by the significant mortality incidence in men.

Concerning Alpha and Omicron waves, no differences by sex in incidence of CV mortality, CV endpoints, or the combined CV endpoint were seen in these waves. Of note, the lowest mortality incidence was observed for patients hospitalized during the Omicron wave: no men and just four (0.67%) women died during 18-month follow-up after hospitalization for SARS-CoV-2-associated pneumonia during Omicron, although the difference was non-significant.

Among the CV endpoints examined, CV hospitalization occurred more often than other endpoints for both sexes during all three waves, and especially during the Delta and Omicron waves (men 11.75% Delta, 11.19% Omicron vs. 5.32% Alpha, *p* <0.001; women 9.35% Delta, 8.17% Omicron versus 4.62% Alpha, *p* <0.001), while other CV endpoints showed no significant differences between the sexes and waves.

**Figure 1:**
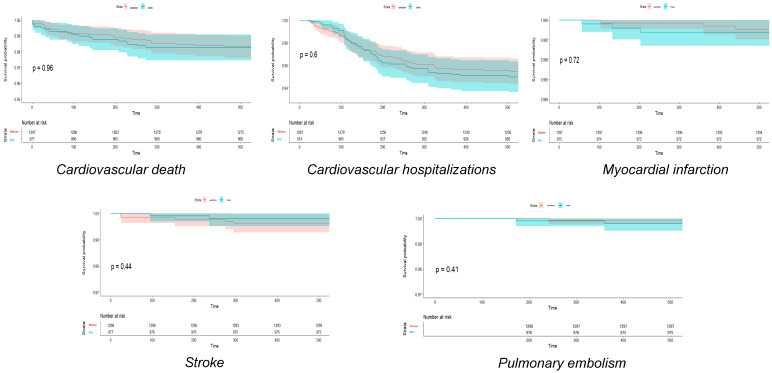
Kaplan-Meier curves and Hexane-Wilcoxon tests of cardiovascular endpoints in Alpha variant regarding to sex.

**Figure 2:**
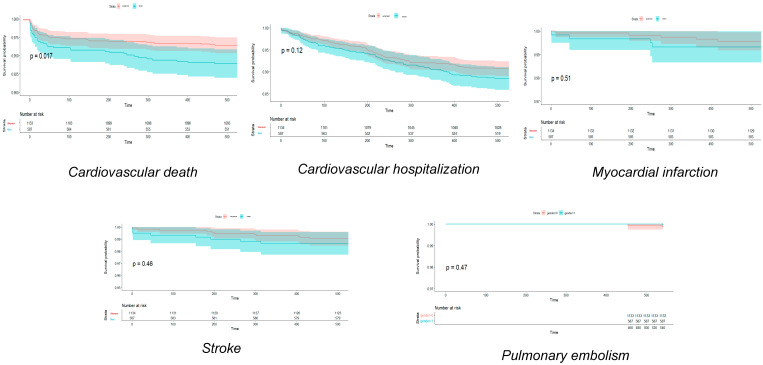
Kaplan-Meier curves and Hexane-Wilcoxon tests of cardiovascular endpoints in Delta variant regarding to sex.

**Figure 3:**
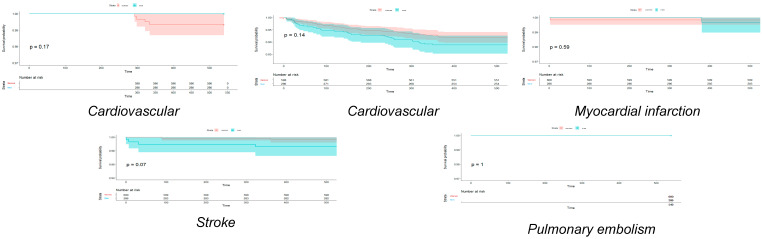
Kaplan-Meier curves and Hexane-Wilcoxon tests of cardiovascular endpoints in Omicron variant regarding to sex.

Figure 1, Figure 2 and Figure 3 show Hexane–Wilcoxon tests and Kaplan–Meier curves depicting survival probabilities by endpoint and by sex according to SARS-CoV-2 variants. In Hexane–Wilcoxon tests presented in Kaplan–Meier curves, there was no significant difference in CV deaths, hospitalizations, MI, stroke, and PE between sexes at 1.5-year follow-up among the Alpha and Delta waves (*p* > 0.05, Figure 1 and Figure 3). In contrast, CV mortality was higher during the Delta variant in men (*p* = 0.017), but no difference was found in the other endpoints by sex in the Delta variant (Figure 2, *p* > 0.05).

To prove the impact of different risk factors, including sex, on CV mortality, separate multivariate Cox proportional hazards regression models were constructed for the endpoint of CV death with a follow-up period of 18 months for the Alpha, Delta, and Omicron waves (see Table 6). According to the likelihood ratio test, the models for CV mortality in Alpha and Delta waves were significant (LR = 50.99, *p* < 0.001) and (LR = 79.49, *p* < 0.001), respectively. The results of the multivariate analysis showed that male gender (HR = 1.676, 95% CI: 1.005–2.796, *p* = 0.048) is a significant risk factor of CV death in FU 18 months after hospitalization only for patients hospitalized with COVID-19 during Delta wave. Age was also a risk factor of CV death for the Alpha (HR = 1.048, 95% CI: 1.018–1.081, *p* = 0.002) and the Delta waves (HR = 1.085, 95% CI: 1.059–1.111, *p* < 0.001), and BMI was also a significant risk factor (HR = 0.905, 95% CI: 0.863–0.949, *p* < 0.001), but only for the Alpha wave. The Cox model of CV mortality for the Omicron wave was constructed on a limited number of endpoints (4 deaths in 18 months FU) and showed limited association with variables and low significance of the model (*p* = 0.09, LR = 9.51). At the same time, the presence of prior myocardial infarction was a significant predictor of survival probability in the Omicron wave (HR = 17.715, 95% CI: 1.138–275.697, *p* = 0.040).

## 4. Discussion

In our study, no significant differences between sexes in the occurrence of cardiovascular endpoints such as CV deaths, CV hospitalizations, MI, stroke, and PE was observed during 18-month follow-up in Alpha and Omicron variants, whereas the Delta variant was associated with higher CV mortality among males during long-term follow-up.

The COVID-19 pandemic has had an inconceivable, worldwide impact on human health and healthcare systems. In the period 2020–2021, the World Health Organization estimated some 287 million confirmed COVID-19 cases worldwide with 5.4 million COVID-19-associated deaths. However, some authors have calculated 14.83 million excess COVID-19 deaths globally, more than 2.74 times the initially reported estimate for the same period [30]. Accurately capturing and reporting infection cases and death rates in every country has been a challenge, especially due to variations in access to testing and differential diagnoses, lack of complete data for all countries, as well as inconsistencies with the certification of COVID-19 as the cause of death [30]. A meta-analysis examining excess COVID-19 mortality in 79 countries between 2020–2022 described a pooled excess mortality rate of 104.84 (95% CI: 85.56–124.13) per 100,000; however, differences by continent and country income status were observed, with South America showing the highest pooled excess mortality (134.01 (95% CI: 68.24–199.8) per 100,000), Oceania the lowest (−32.15% (95% CI: −60.53–−3.77)), and developing countries showing higher excess mortality than in developed countries (135.8 (95% CI: 107.83–163.76) versus 68.08 (95% CI: 42.61–93.55), respectively) [31]. In the same meta-analysis, males showed higher pooled excess mortality (130.10 (95% CI: 94.15–166.05)) compared to females (102.16 (95% CI: 85.76–118.56)) and authors noted the highest pooled excess mortality occurring in the older population age ≥ 60 years (781.74 (95% CI: 626.24–937.24)) [31].

Emerging COVID-19 data have highlighted the existence of some sex disparities in infectious disease outcomes. Indeed, higher incidence and increased fatality rates have been observed in males compared to females during previous human coronavirus outbreaks, such as SARS-CoV-1 and Middle Eastern Respiratory Syndrome coronavirus (MERS-CoV) [32]. A meta-analysis of 59 COVID-19 studies comprising 36,000 patients showed that men had a higher risk of infection, disease severity, intensive care unit admission, and death than women [33]. In another study, men had a statistically significant 8% higher risk of being diagnosed with COVID-19 than women [33]. In a meta-analysis of 3,111,714 patients from 46 countries, males were more likely than females to require intensive care unit admission (odds ratio (OR) = 2.84, 95% confidence interval (CI) = 2.06–3.92, *p* = 1.86 × 10^−10^) and to succumb to SARS-CoV-2 infection (OR = 1.39, 95% CI = 1.31–1.47, *p* = 5 × 10^−30^) [34].

A Hungarian study observed age and sex differences in the excess all-cause mortality between the second and third COVID-19 waves, noting significantly higher excess mortality in all ages groups in both the second and third waves compared to pre-pandemic control groups, with the exception of non-significant excess for the age 0–34 population and, in the third wave, for the 85+ population [35]. Authors linked the reduced relative mortality among oldest adults during the third wave to the prioritization of the elderly in immunization programs. Concerning sex differences in excess all-cause mortality, only a higher rate ratio of mortality in women aged 55–64 compared to men during the third wave of the pandemic was observed; otherwise, no significant differences for other age groups and waves by sex were seen [35]. An Italian study evaluated the effect of sex, age, and period of infection on the overall survival of hospitalized and non-hospitalized COVID-19 patients in three waves. In the first wave of the pandemic, age and male sex were highly significant independent risk factors for death, with sharp mortality increase for infected patients ≥ 65 years and tremendously high risk for patients ≥ 85 years recorded. Male sex was associated with a twofold risk of death (HR = 2.05) compared to females in the first wave of the pandemic [36]. The first pandemic wave was associated with a threefold increased risk of death compared to subsequent periods (HR = 0.379 and 0.46 in the second and third waves, respectively). Among hospitalized patients, age, male sex, and pandemic wave were significantly associated with risk of death 30 days after hospitalization; however, following the first wave, the risk of death was diminished by roughly 30% in subsequent waves [36]. Male sex was confirmed to be a prognostic factor for death in both hospitalized and non-hospitalized patients to day 30 [36].

Several mechanisms have been attributed to the observed sex differences in susceptibility, severity of infection, and COVID-19 mortality rates. Women tend to show a more robust immune response to viruses and vaccines and have better abilities for immune-mediated tissue repair. Additionally, men and women show different circulation levels of angiotensin-converting enzyme 2 (ACE2), the principal entry receptor for SARS-CoV-2 that is expressed in numerous tissues such as nasal, respiratory, and intestinal epithelial cells. Estrogen down-regulates ACE2, providing women with lower expression and a potential immune system advantage [37,38]. Men also have a higher expression of the mucosa-specific serine protease TMPRSS2, which enables virus entry into host cells; androgens upregulate TMPRSS2 expression, thus delaying virus clearance in males [37,38]. Innate and adaptive immune responses are more developed in women, including the more robust Type 1 IFN responses necessary for restriction of viral replication and a higher ratio of CD3- and CD4- T cells, which drive cellular immunity in females [23]. Estrogen not only stimulates higher T cell response in women, it also contributes to vasodilation and inhibition of viral replication through catalysis of nitric oxide from L-arginine [23]. Sex-specific immune response is less robust in men who demonstrate poorer T-cell response and increased inflammatory cytokine levels of IL-8 and IL-18 [23]. Mourosi et al. describe the role of non-coding RNA in the regulation of the immune system, elucidating differences in the expression of microRNA and long non-coding RNA occurring between men and women. MicroRNA modulates antiviral immunity during viral infection. While the X chromosome contains some 10% of microRNA in the human genome, the Y chromosome contains only 2 microRNAs, potentially disadvantaging males [23].

Comparative severity of the most widespread European COVID-19 variants Alpha, Delta, and Omicron has been examined in multiple studies using hospital admission, ICU admission, or death within 28 days of a positive COVID-19 test as outcomes. Severity differed by variant, country, and vaccination rate; however, the Delta variant has been described as the most severe in several studies [39,40,41,42]. The Delta variant, which was most widespread in 2022, demonstrated the highest mortality in the acute period [42] and was also shown as the most severe concerning cardiovascular mortality and other cardiovascular endpoints in the post-COVID period [43]. Despite this, sex-disaggregated data evaluating the severity of outcomes by variants remain limited.

In our study, we investigated 4882 hospitalized patients with moderate to severe SARS-CoV-2-associated pneumonia and followed them for 18 months after hospital discharge. As the patients were enrolled between April 2020 and April 2022, they were distributed according to COVID-19 variants of concern, Alpha, Delta, and Omicron, and sex-disaggregated data were analyzed. Patients were younger in the Alpha group and had higher BMI. However, in the Delta group, patients more frequently demonstrated concomitant illnesses such as DM2, CHD, prior stroke, prior MI, and COPD, yet the number of comorbidities aligned with those described in other non-COVID studies. According to some studies, comorbidities such as hypertension, diabetes, cardiovascular disease, and chronic lung diseases are highly prevalent among COVID-19 fatalities [44]. The interaction between sex, comorbidities, and COVID-19 severity, however, is unclear [44]. Among older individuals, multiple morbidities are described as more commonly occurring in women (45%) compared to men (32%) [45], yet other studies describe a higher prevalence of comorbidities, such as hypertension, diabetes, and positive smoking status, occurring among men [46].

Sex differences have been demonstrated in multiple COVID-19-related investigations, especially in the acute phase of infection and during post-acute follow-up. Morbidity and hospital mortality were higher in men in the acute phase of infection, as described in numerous studies [10,14,15,25]. Epidemiological studies from 38 countries reported a mean case fatality rate (CFR) in males that was 1.7 times higher than the mean CFR in females, with advancing age described as a risk factor for mortality in both sexes [47]. In a large study of 17 million adults in the UK, a significant correlation between male sex and risk of COVID-19-associated mortality, with a hazard ratio of 1.59, was observed. Thus, current epidemiological evidence suggests a male bias with respect to greater susceptibility to SARS-CoV-2 infection and severe COVID-19 clinical outcomes [12].

However, PACS or long COVID is more frequent in women than in men [15]. Female sex and 40–50 years of age are considered risk factors for long COVID, suggesting a possible sex hormone link at the time of perimenopause or long COVID and menopause symptom overlap [48,49]. While the long-lasting symptoms of most other diseases in adults are primarily associated with severe illness following longer hospitalization at an ICU and with older age and more comorbidities, most long COVID-19 patients initially had a mild to moderate course of infection without requiring lengthy hospitalization and were largely unaffected by preexisting conditions [48,49]. The underlying mechanisms behind this inverse effect in long COVID-19 are not entirely clear. The described outcome disparities between men and women, however, illustrates the need for further outcomes investigation and sex-disaggregated data analysis.

In our study, more women were hospitalized with moderate to severe COVID-19-associated pneumonia during all COVID-19 variant waves: 57.1% of hospitalized COVID-19 patients were women during the Alpha wave, 65.9% women during the Delta wave, and 67.7% during the Omicron wave. Hospitalization among men by number of patients was highest at the beginning of the pandemic (Alpha) and decreased steadily during later variants (from 42.9% in Alpha to 32.3% during Omicron), while incidence in women increased in subsequent waves. Men had a trend toward lower BMI in all waves (*p* > 0.05). Men were also younger than women in the Alpha and Delta waves, but not during Omicron (*p* = 0.049). As for concomitant diseases, men more often had CHD, prior MI, and COPD in Alpha, but women had higher rates of arterial hypertension (*p* < 0.001). In Delta, CHD, prior MI, and stroke were the more frequently observed comorbidities among men (*p* < 0.001). During the Omicron wave, the comorbidities heart failure (*p* = 0.002) and stroke (*p* = 0.001) were more common among men than women hospitalized for COVID-19-associated pneumonia. Such disparities underline the features of COVID-19 variants and potential sex-dependent responses to COVID-19 variants.

It has been suggested that the males may be more predisposed to COVID-19 infection compared to females. Male predisposition has been attributed to the different effects of hormones in inflammatory processes, differences in levels of cell receptors (angiotensin-converting enzyme), and molecules that facilitate SARS-CoV-2 entry through virus-cell membrane fusion (transmembrane protease serine 2, TMPRSS2) [16,23]. Analysis of plasma and circulating immune cells from male and female patients with COVID-19 identified distinct factors associated with disease progression in each sex, suggesting that sex-specific variability in the immune response may contribute to differences in COVID-19 outcomes [16]. However, male predisposition to COVID-19 infection may also be attributed to gender differences driven by social and cultural norms such as lifestyle differences (i.e., men shown to wash hands less frequently or to isolate less), risk-associated behaviors (i.e., higher smoking, drinking rates among men), or access to healthcare (i.e., access to testing, access to hospital care differing by gender) [16].

In our study, CV mortality during 18-month FU was observed in 2.48% of the total cohort and was highest during the Delta wave (4.47%) and lowest during Omicron (0.45%) and is thus comparable to usual non-COVID virus infections. CV hospitalization rates during the Delta (10.17%) and Omicron (9.14%) waves were higher than those observed during the Alpha wave (4.92%). Stroke and combined CV endpoints also differed significantly between the three variants, more often occurring among patients infected during the Delta wave. As for potential sex differences in CV endpoints occurring during the 18-month follow-up in our study, none were observed between the sexes during the Alpha and Omicron waves (*p* > 0.05), yet CV mortality was significantly higher for men (*p* = 0.017) during the Delta wave. Mirroring this finding, the combined CV endpoint (*p* = 0.017) was also higher for men during the Delta wave.

In our study, the incidence of CV hospitalizations occurring over long-term 18-month follow-up persisted between Delta and Omicron variants, especially among men (11.75% and 11.19%, respectively). For clinicians, screening of patients following hospitalization for COVID-19 infection is warranted to evaluate for potential late CV complications and prevent further events, especially in patients with severe courses of infection, those experiencing acute CV manifestations during infection, those with preexisting CV disease, multi-morbid patients, those with risk factors for cardiovascular disease (i.e., hypertension or diabetes), and those with the presence of new or ongoing symptoms (i.e., arrythmias, hypercoagulability, persisting inflammatory state) [50]. While the incidence of patients experiencing CV events such as myocardial infarction, stroke, or PE during long-term follow-up is relatively low, in single digits, as demonstrated in our study, there is still potential for large numbers of patients to experience CV events given widespread COVID-19 infection at the population level.

In our study, differences in demographic data and concomitant diseases between the COVID-19 variants of concern could be identified. The largest number of CV events, including CV mortality, during an 18-month follow-up period occurred during the Delta variant. Among men, significant differences between the three variants were observed in CV mortality, CV hospitalization, and the combined CV endpoint (all *p* < 0.001), with higher incidences among men observed during the Delta wave. Among women, significant differences between the three waves were also observed for CV mortality, CV hospitalizations, and the combined CV endpoint (all *p* < 0.001), with the highest incidence occurring during the Delta wave. The lowest number and percentage of CV events occurred during Omicron wave. Our findings align with other studies describing higher severity and higher incidence of CV outcomes occurring during the Delta variant when compared to the Omicron variant [42].

### Limitation of the Study

A few limitations in our study must be reported. Despite the large sample size, the study was single center, and thus limited in its findings to a specific region within the unique dynamic context of the pandemic and evolution of variants in that area. Another limitation concerns the classification of variants: only some, but not all, patients were virologically identified using a smear test. The vaccination status was not captured in all patients and not analyzed, yet is a factor potentially impacting the incidence of cardiovascular outcomes.

## 5. Conclusions

Our study of a large cohort of 4882 patients hospitalized with acute moderate to severe COVID-19-associated pneumonia evaluated potential sex differences in CV mortality and CV outcomes over a long-term, 18-month follow-up period, additionally analyzing if Alpha, Delta, and Omicron COVID-19 variants had potential negative impacts on CV mortality and CV outcomes by sex. The only difference observed between sexes was the significantly higher incidence of CV death among men during the Delta wave, as well as a corresponding significantly higher incidence of the combined CV endpoint among men during the Delta wave. Significant differences in CV mortality, CV hospitalization, and the combined CV endpoint could be seen between the waves within the separate groups of males and females. The highest incidence of CV events, with the exception of PE, occurred during the Delta variant, with the lowest incidence of CV endpoints occurring during Omicron. This study provided valuable insights into the incidence of long-term CV outcomes following COVID-19 infection by waves as well as sex-disaggregated data analysis in a large post-COVID-19 cohort.

## Figures and Tables

**Table 1:** Clinical-demographic characteristics of the total COVID-19 patient population by variant.

	All	Alpha	Delta	Omicron	Compare
*n*	4882				
Sex, m/f, *n* (%)	1850/3032 (61.0/39.0)	977/1298 (75.3/24.7)	587/1134 (51.8/48.2)	286/600 (47.7/52.3)	χ^2^ = 58.28 *** p < 0.001
Age, *n* (%)	59.2 (46.25; 67.71)	57.26 (47.42; 66.57)	59.66 (46.66; 66.66)	63.3 (42.48; 72.7)	Z = 4.18 *** p < 0.001
BMI, *n* (%)	27.5 (24.41; 30.64)	28.14 (25.16; 30.78)	27.92 (24.65; 31.64)	25.05 (22.03; 28.35)	Z = 3.63 *** p < 0.001
Hypertension, *n* (%)	2189 (44.83%)	871 (38.29%)	969 (56.31%)	349 (39.39%)	χ^2^ = 141.61 *** p < 0.001
DM2, *n* (%)	599 (12.27%)	269 (11.82%)	233 (13.54%)	97 (10.95%)	χ^2^ = 4.13 *p* = 0.109
CKD, *n* (%)	115 (2.36%)	84 (3.69%)	15 (0.87%)	16 (1.81%)	χ^2^ = 35.32 *** p < 0.001
CHD, *n* (%)	624 (12.78%)	210 (9.23%)	328 (19.06%)	86 (9.71%)	χ^2^ = 94.07 *** p < 0.001
Prior MI, *n* (%)	152 (3.11%)	64 (2.81%)	64 (3.72%)	24 (2.71%)	χ^2^ = 3.25 *p* = 0.197
Prior Stroke, *n* (%)	113 (2.31%)	40 (1.75%)	60 (3.49%)	13 (1.47%)	χ^2^ = 16.38 *** p < 0.001
Heart Failure, *n* (%)	315 (6.45%)	189 (8.31%)	100 (5.81%)	26 (2.93%)	χ^2^ = 32.31 *** p < 0.001
COPD, *n* (%)	214 (4.38%)	75 (3.29%)	99 (5.75%)	40 (4.52%)	χ^2^ = 14.14 *** p < 0.001

Legend: BMI—body mass index, MI—myocardial infarction. ***—differences and significance according to Kruskal-Wallis test for continuous variables and χ^2^ for factor variables at *p* < 0.001.

**Table 2:** Comparison of sex/BMI/age characteristics of COVID-19 patients by variant.

	Sex, Median (Q1; Q3)	BMI, Median (Q1; Q3)	Age, Median (Q1; Q3)
Alpha	Men	27.76 (25.18; 28.57)	56 (44; 66)
	Women	28.42 (25.15; 32.44)	58.22 (50; 67)
	P-test Mann-Whitney	0.059	<0.001 ***
Delta	Men	27.68(24.74; 30.86)	59 (46; 66)
	Women	28.04 (24.61; 32.05)	60 (47; 67)
	P-test Mann-Whitney	0.106	0.186
Omicron	Men	24.17 (22.13; 27.75)	64 (53; 72)
	Women	25.43 (21.99; 28.6)	63 (38; 73)
	P-test Mann-Whitney	0.957	0.049 *

Legend: *, ***—difference according to Mann-Whitney test, *p* < 0.05 and *p* < 0.001, respectively.

**Table 3:** Comparison of concomitant diseases of COVID-19 patients by sex and variant.

Xi-Quadrat								
m/w	AH	DM2	CKD	CHD	Prior MI	Heart Failure	Prior Stoke	COPD
Alpha, *n* = 2275								
Men, *n* = 977	322 (32.96%)	99(10.13%)	28 (2.87%)	118 (12.08%)	44 (4.5%)	98 (10.03%)	20 (2.05%)	47 (7.68%)
Women, *n* = 1298	549 (42.30%)	170 (13.09%)	56 (4.31%)	92 (7.09%)	20 (1.54%)	91 (7.01%)	20 (1.54%)	28 (6.78%)
*p* (m/f)	χ^2^ = 20.57 *** p < 0.001	χ^2^ = 4.697 * p = 0.031	χ^2^ = 3.289 *p* = 0.070	χ^2^ = 16.57 *** p < 0.001	χ^2^ = 17.89 *** p < 0.001	χ^2^ = 6.674 * p = 0.010	χ^2^ = 0.827 *p* = 0.364	χ^2^ = 12.31 *** p < 0.001
Delta, *n* = 1721								
Men, *n* = 587	320 (54.51%)	71 (12.09%)	7 (1.19%)	131 (22.32%)	34 (5.79%)	37 (6.30%)	34 (5.79%)	42 (7.16%)
Women, *n* = 1134	649 (57.23)	162 (14.29%)	8 (0.71%)	197 (17.37%)	30 (2.65%)	63 (5.569%)	26 (2.29%)	57 (5.03%)
*p* (m/w)	χ^2^ = 1.160 *p* = 0.282	χ^2^ = 1.585 *p* = 0.209	χ^2^ = 1.052 *p* = 0.303	χ^2^ = 6.130 *p* = 0.014 *	χ^2^ = 10.69 *p* = 0.002 **	χ^2^ = 0.395 *p* = 0.530	χ^2^ = 14.08 *** p < 0.001	χ^2^ = 3.232 *p* = 0.073
Omicron, *n* = 886								
Men, *n* = 286	115 (40.21%)	30 (10.49%)	5 (1.75%)	34 (11.89%)	10 (3.49%)	16 (5.59%)	10 (3.49%)	18 (6.29%)
Women, *n* = 600	234 (39%)	67 (11.17%)	11 (1.83%)	52 (8.67%)	14 (2.33%)	10 (1.67%)	3 (0.5%)	22 (3.67%)
*p* (m/w)	χ^2^ = 0.119 *p* = 0.731	χ^2^ = 0.091 *p* = 0.763	χ^2^ = 0.008 *p* = 0.930	χ^2^ = 2.293 *p* = 0.130	χ^2^ = 0.994 *p* = 0.319	χ^2^ = 10.49 *p* = 0.002 **	χ^2^ = 12.03 *** p < 0.001	χ^2^ = 3.101 *p* = 0.079
3 waves comparison (Fisher criteria)								
Men (Alpha-Delta-Omicron)	χ^2^ = 70.55 *** p < 0.001	χ^2^ = 1.501 *p* = 0.473	χ^2^ = 5.128 *p* = 0.077	χ^2^ = 32.70 *** p < 0.001	χ^2^ = 2.530 *p* = 0.283	χ^2^ = 9.77 ** p = 0.008	χ^2^ = 15.04 *** p < 0.001	χ^2^ = 3.86 *p* = 0.146
Women (Alpha-Delta-Omicron)	χ^2^ = 74.49 *** p < 0.001	χ^2^ = 3.348 *p* = 0.188	χ^2^ = 33.94 *** p < 0.001	χ^2^ = 69.12 *** p < 0.001	χ^2^ = 3.754 *p* = 0.154	χ^2^ = 21.22 *** p < 0.001	χ^2^ = 24.33 *** p < 0.001	χ^2^ = 8.013 * p = 0.019

*, **, ***—difference according to Xi-quadrat at *p* < 0.05, *p* < 0.01 and *p* < 0.001, respectively.

**Table 4:** Cardiovascular endpoints in COVID-19 patients at 18 months post-hospitalization by variant.

	All	Alpha	Delta	Omicron	Compare
*n*	4882	2275	1721	886	
Cardiovascular death	121 (2.48%)	40 (1.76%)	77 (4.47%)	4 (0.45%)	χ^2^ = 48.31 *** p < 0.001
Cardiovascular hospitalizations	368 (7.53%)	112 (4.92%)	175 (10.17%)	81 (9.14%)	χ^2^ = 42.67 *** p < 0.001
MI	20 (0.41%)	9 (0.4%)	9 (0.52%)	2 (0.23%)	χ^2^ = 1.29 *p* < =0.526
Stroke	32 (0.66%)	7 (0.31%)	19 (1.1%)	6 (0.68%)	χ^2^ = 9.55 ** p = 0.009
PE	4 (0.08%)	3 (0.13%)	1 (0.06%)	0 (0%)	χ^2^ = 1.54 *p* < =0.464
Combined endpoint	493 (10.1%)	163 (7.17%)	242 (14.06%)	88 (9.93%)	χ^2^ = 51.37 *** p < 0.001

Legend: MI—myocardial infarction, PE—pulmonary embolism. **, ***—different according to Xi-quardat at *p* < 0.01 and *p* < 0.001, respectively.

**Table 5:** Comparison of cardiovascular endpoints in COVID-19 patients at 18 months post-hospitalization by sex and variant.

Xi-Quadrat						
m/w	Endpoints					
	Cardiovascular Death	Cardiovascular Hospitalizations	Myocardial Infarction	Stroke	PE	Combined Endpoint
Alpha, *n* = 2275						
Men, *n* = 977	17 (1.74%)	52 (5.32%)	5 (0.51%)	2 (0.21%)	2 (0.21%)	75 (7.68%)
Women, *n* = 1298	23 (1.77%)	60 (4.62%)	4 (0.31%)	5 (0.39%)	1 (0.08%)	88 (6.78%)
*p* (m/w)	χ^2^ = 0.003 *p* = 0.955	χ^2^ = 0.583 *p* = 0.445	χ^2^ = 0.184 ^ǂ^ *p* = 0.669	χ^2^ = 0.150 ^ǂ^ *p* = 0.699	χ^2^ = 0.061 ^ǂ^ *p* = 0.805	χ^2^ = 0.674 *p* = 0.412
Delta, *n* = 1721						
Men, *n* = 587	36 (6.13%)	69 (11.75%)	4 (0.68%)	8 (1.36%)	0 (0%)	99 (16.87%)
Women, *n* = 1134	41 (3.62%)	106 (9.35%)	5 (0.44%)	11 (0.97%)	1 (0.09%)	143 (12.61%)
*p* (m/w)	χ^2^ = 5.735 *p* = 0.017 *	χ^2^ = 2.454 *p* = 0.118	χ^2^ = 0.430 ^ǂ^ *p* = 0.512	χ^2^ = 0.547 *p* = 0.460	χ^2^ = 0.112 ^ǂ^ *p* = 0.778	χ^2^ = 5.795 *p* = 0.017 *
Omicron, *n* = 886						
Men, *n* = 286	0 (0%)	32 (11.19%)	1 (0.35%)	4 (1.4%)	0 (0%)	35 (12.24%)
Women, *n* = 600	4 (0.67%)	49 (8.17%)	1 (0.17%)	2 (0.33%)	0 (0%)	53 (8.83%)
*p* (m/w)	χ^2^ = 1.915 *p* = 0.167	χ^2^ = 2.130 *p* = 0.145	χ^2^ = 0.049 ^ǂ^ *p* = 0.826	χ^2^ = 1.876 *p* = 0.171	χ^2^ = 0 *p* = 1	χ^2^ = 2.509 *p* = 0.114
3 waves comparison (Fisher criteria)						
Men (Alpha-Delta-Omicron)	χ^2^ = 35.41 *** p < 0.001	χ^2^ = 23.79 *** p < 0.001	χ^2^ = 0.426 *p* = 0.809	χ^2^ = 8.41 * p = 0.015	χ^2^ = 1.709 *p* = 0.409	χ^2^ = 31.195 *** p < 0.001
Women (Alpha-Delta-Omicron)	χ^2^ = 21.31 *** p < 0.001	χ^2^ = 21.82 *** p < 0.001	χ^2^ = 0.926 *p* = 0.630	χ^2^ = 4.349 *p* = 0.114	χ^2^ = 2.299 *p* = 0.317	χ^2^ = 24.33 *** p < 0.001

^ǂ^—with Yats equation, *, ***—difference according to Xi-quadrat at *p* < 0.05 and *p* < 0.001, respectively.

**Table 6:** Multivariate model of CV mortality in three COVID-19 waves.

Variables	HR (95% CI), *p*-Level		
	Alpha	Delta	Omicron
Sex (male)	0.967 (0.479–1.948) *p* = 0.925	1.676 (1.005–2.796) *p* = 0.048 *	–
Age, year	1.048 (1.018–1.081) *p* = 0.002 **	1.085 (1.059–1.111) *p* < 0.001 ***	1.045 (0.981–1.114) *p* = 0.173
BMI	0.905 (0.863–0.949) *p* < 0.001 ***	1.019 (0.971–1.069) *p* = 0.448	1.058 (0.972–1.150) *p* = 0.189
AH	0.775 (0.358–1.677) *p* = 0.517	0.531 (0.202–1.397) *p* = 0.520	0.199 (0.014–2.941) *p* = 0.099
DM	1.241 (0.501–3.076) *p* = 0.640	1.624 (0.886–2.977) *p* = 0.116	–
CKD	2.029 (0.702–5.868) *p* = 0.192	1.609 (0.358–7.232) *p* = 0.535	–
CHD	0.929 (0.181–4.768) *p* = 0.929	1.105 (0.608–2.009) *p* = 0.743	–
Post MI	0.208 (0.026–1.642) *p* = 0.136	1.994 (0.839–4.736) *p* = 0.118	17.715 (1.138–275.697) *p* = 0.040 *
Heart failure	4.754 (0.944–23.949) *p* = 0.059	0.683 (0.254–1.837) *p* = 0.449	–
Post stroke	1.674 (0.369–7.585) *p* = 0.504	1.666 (0.679–4.084) *p* = 0.265	–
COPD	0.709 (0.148–3.396) *p* = 0.667	1.712 (0.824–3.559) *p* = 0.149	–
LR, *p*-level Concordance ± SE	50.99, *p* < 0.001 *** 0.794 ± 0.04	79.49, *p* < 0.001 *** 0.804 ± 0.04	9.51, *p* = 0.09 0.918 ± 0.032

Legend: *, **, ***—coefficients in risk factors at *p* < 0.05, *p* < 0.01 and *p* < 0.001, respectively.

## Data Availability

Data available upon request due to ethical reasons (data protection rules). Please contact first and last authors: k.kopp@salk.at and znaufal@mail.ru.
